# 540. Does *Remdesivir* Impact the Clinical Outcome of Patients with *COVID 19* Infection?

**DOI:** 10.1093/ofid/ofab466.739

**Published:** 2021-12-04

**Authors:** Karthik Gunasekaran, Jisha S John, Hanna Alexander, Naveena Gracelin, Prasanna Samuel, Priscilla Rupali

**Affiliations:** Christian Medical College, Vellore, Vellore, Tamil Nadu, India

## Abstract

**Background:**

Remdesivir (RDV), was included for the treatment of mild to moderate COVID-19 since July 2020 in our institution, following the initial results from ACTT-1 interim analysis report. With the adoption of RDV, there seems to be anecdotal evidence of efficacy as evidenced by early fever defervescence, quick recovery when on oxygen with decreased need for ventilation and ICU care. We aimed to study the impact of RDV on clinical outcomes among patients with moderate to severe COVID –19.

**Methods:**

Nested case control study in the cohort of consecutive patients with moderate to severe COVID – 19. Cases were patients initiated on RDV and age and sex- matched controls who did not receive RDV were included. The primary outcome was in-hospital mortality. Secondary outcomes were, duration of hospital stay, need for ICU, duration of oxygen therapy and need for ventilation.

**Results:**

A total of 926 consecutive patients with COVID – 19 were included, among which 411 patients were cases and 515 controls. The mean age of the cohort was 57.05±13.5 years, with male preponderance (75.92%). The overall in-hospital mortality was 22.46%(n=208). On comparison between cases and controls there was no statistically significant difference with respect to primary outcome [22.54% vs. 20.78%, (p value: 0.17)]. Progression to non-invasive ventilation (NIV) was higher among the controls [24.09% vs. 40.78% (p value: < 0.001*)]. Progression to invasive ventilation was also higher among the controls [5.35% vs. 9.71% (p value: 0.014*)]. In subgroup analysis among critically ill patients, the use of RDV showed decrease in mortality (OR 0.32 95% CI; 0.13 – 0.75 p value – 0.009*).

**Conclusion:**

RDV did not decrease the in-hospital mortality among moderate to severe COVID – 19. However, there seems to be a significant reduction in mortality in critically ill patients.

**Disclosures:**

**All Authors**: No reported disclosures

